# The Antecedents of Thriving at Work: A Meta-Analytic Review

**DOI:** 10.3389/fpsyg.2021.659072

**Published:** 2021-08-05

**Authors:** Danping Liu, Siwen Zhang, Yanling Wang, Yufei Yan

**Affiliations:** ^1^School of Management, Xihua University, Chengdu, China; ^2^Research Institute of International Economics and Management, Xihua University, Chengdu, China; ^3^Darla Moore School of Business, University of South Carolina, Columbia, SC, United States; ^4^Business School, Yunnan University of Finance and Economics, Kunming, China; ^5^School of Business Administration, Southwestern University of Finance and Economics, Chengdu, China

**Keywords:** thriving at work, antecedents, unit contextual features, resources produced at work, individual agentic work behaviors, personality traits, national culture

## Abstract

In this study, a systematic and comprehensive meta-analysis of the relationship between thriving at work and its antecedents is conducted. The antecedents in terms of the characteristics of unit contextual features, the resources produced at work, agentic work behaviors, and personality traits are illustrated according to the socially embedded model of thriving described by Spreitzer and research. Additionally, we examine possible cultural influence on the relationship between thriving and its antecedents at different levels of individualistic culture. According to 67 independent samples (*N* = 28,097), our findings reveal the correlations between thriving at work and the antecedents such as those in the form of unit contextual features, the resources produced at work, agentic work behaviors, and personality traits. Furthermore, we find that individualism moderate the relationships between certain antecedents and thriving at work. Finally, we discuss the theoretical and practical implications of this study as well as the directions for future research.

## Introduction

Being a positive psychological state, thriving at work has attracted increased attention in recent decades. It is defined as “the joint experience of a sense of vitality and learning and is most accurately conceptualized as a continuum—where people are more or less thriving at any point in time—rather than a dichotomous state of either thriving or not” (Porath et al., 2012, p. 250). Thriving is extremely important, because vitality and learning are the two key factors in improving performance and well-being, to talented employees who face intensifying competitions in the workplace (Pfeffer, 2010).

Several scholars and managers have paid attention to thriving at work due to its positive effects. A few studies have shown that thriving at work contributes to the improvement of task performance, job satisfaction, and physical well-being (Spreitzer et al., 2012). Furthermore, Porath et al. (2012) proposed that thriving was a different construct when compared to a positive/negative effect and learning goal orientation, etc., and found this notion, which in turn could better explain these outcomes. Compared with employees who fail to be in the state of thriving, employees who are in the state of thriving at work can continue to acquire growth and self-development, which ultimately promote organizational efficiency and prosperity (Han and Wei, 2013). In addition, employees are more likely to have negative emotions due to an increasing competition in the workplace, which induces many problems such as burnout, the lack of work vitality, and even poor health conditions. Therefore, it is always the pursue for managers to keep employees vigorous and efficient at work. In sight of the importance of thriving in the workplace, it is critical to explore the factors that motivate employees’ thriving at work.

Many factors, including organizational context, job characteristics, and personal factors, have an impact on employees’ thriving at work such as perceived organizational support (Collins, 2014), trust (Carmeli et al., 2009), workplace incivility (Panagiotis et al., 2013), job crafting (Han, 2017), challenge stress (Flinchbaugh et al., 2015), and proactive personality (Albi et al., 2020). Recently, some other constructs have served as important antecedents of employees’ thriving at work such as workplace friendship (Chen et al., 2016), taking charge (Xu et al., 2020), and social functioning (Zhang et al., 2020). Despite these profound research findings, some inconsistent conclusions were obtained. For example, Jaiswal and Dhar (2017) found that work experience was one of the important drivers of thriving at work. However, it was confirmed by Zhang R. G. et al. (2018) that individuals with more work experience were lacking in thriving at work. Such a discrepancy probably arises due to the accuracy of statistical results being subjected to the possible measurement and sampling error and the moderating role of some contingent variables. Therefore, a meta-analysis is needed to address this problem by integrating the existing results in empirical studies by controlling both kinds of statistical errors and considering some contextual factors.

By systematically examining the antecedents of the characteristics of individuals and relational resources, the meta-analysis mentioned in Kleine et al. (2019) provided a general research framework according to the socially embedded model of thriving (Spreitzer et al., 2005). Despite being influential, their study excluded some other prominent categories of indicators such as unit contextual features and individual agentic work behaviors. Simultaneously, a greater number of indicators in each category of antecedents are needed to be further explored, thus necessitating a more in-depth exploration and finely grained meta-analytic review of the antecedents that engender thriving.

Furthermore, the impact of culture on the relationship between thriving and its antecedents requires investigation, ensuing the potential variations in the relationships among various indicators and thriving, identified in a previous study, as existing across cultural contexts (Rozkwitalska, 2018). Specifically, according to the socially embedded model of thriving at work (Spreitzer et al., 2005), the personal development of employees depends heavily on dynamic interactions with others, which are assumed to occur in different patterns, in various cultural contexts. However, the effects of these different patterns in different cultures, on various indicators and thriving, are not yet known.

This study contributes to this field in several ways. Firstly, we have extended the research of Kleine et al. (2019) by integrating additional categories of antecedents into the existing research model. We meta-analyzed the relationship between unit contextual features and thriving, such as challenge stressors, hindrance stressors, work control, job crafting, and organizational justice, which is not noted in their study. We also performed a meta-analysis of the relationship between thriving at work and individual agentic work behaviors such as task focus, exploration, and heedful relating. Secondly, to supplement the study by Kleine et al. (2019) on resources and personality traits (antecedents), we conducted a review of several types of resources produced at work, which promote employees’ thriving, including types of leadership, positive meaning, and work experience. A further meta-analysis of four additional types of personality traits, including self-efficacy (Geiger, 2013), optimism, openness, and conscientiousness, was also performed. Thirdly, we discuss the differences in the relationships between thriving and its antecedents across employees from different cultures. Specifically, we examine the moderating effects of individualistic culture on the relationship between thriving and its antecedents.

## Theoretical Background and Hypotheses

### Theoretical Backgrounds

Previous empirical studies have paid close attention to the antecedents of employees’ thriving at work. Some studies explored the motivators who enable employees to thrive at work from the perspective of the characteristics of individuals such as psychological capital (Paterson et al., 2014), proactive personality (Albi et al., 2020), and positive affect (Porath et al., 2012). Furthermore, a series of empirical studies have been carried out for studying the impact of organizational context on employees’ thriving at work. For instance, the existing research covers leadership style (Russo et al., 2018), managerial coaching (Raza and Ahmed, 2020), fairness perception (Ghulam et al., 2020), etc. Other studies focus on job characteristics, including innovation and feedback (Xie, 2016), decision-making discretion (Liu and Bern-Klug, 2013), challenging stress (Prem et al., 2017), etc. Meanwhile, some researchers explored the effects of some antecedents on employees’ thriving at work from the perspective of workplace interpersonal relationship. The existing results show that workplace friendship (Chen et al., 2016), colleague relationship (Xie, 2016; Ehrhardt and Ragins, 2019), workplace incivility (Gkorezis et al., 2013), and knowledge hiding (Jiang et al., 2019) will also affect employees’ thriving at work in different ways.

As shown in Table 1, although they were not exhaustive, the major determinants of thriving at work were summarized through an in-depth review of its antecedents. Because a number of fruitful and influential achievements have been made on the topic of employees’ thriving at work in these years, it is necessary to systematically review and sort out the scattered research, not only to reflect on its omissions and weaknesses but also to make forward-looking prospects for future research.

**TABLE 1:** Summary of the antecedents of thriving at work.

Antecedents	References
Abusive supervision	Liu (2016), Luo (2016), Zhao et al. (2018), Usman et al. (2021)
Authentic leadership	An (2015), Mortier et al. (2016), Xu et al. (2017), Shen et al. (2018)
Autonomy	Gu (2015), Xie (2016), Li (2018)
Broad information sharing	Liu and Bern-Klug (2013)
Challenge stress	Flinchbaugh et al. (2015), Prem et al. (2017)
Conscientiousness	Hennekam (2017)
Decision-making discretion	Liu and Bern-Klug (2013), Sia and Duari (2018), Novaes et al. (2020)
Empowering leadership	Li et al. (2016), Han (2017), Ali et al. (2018)
Exploration	Sia and Duari (2018)
Fairness perception	Ghulam et al. (2020)
Feedback	Lee et al. (2015), Xie (2016)
Heedful relating	Niessen et al. (2012), Ted et al. (2013), Paterson et al. (2014), Abid et al. (2016), Sia and Duari (2018)
High-performance work system	Liu (2017), Zhang et al., (2018)
Job crafting	Han (2017), Wang (2018)
Job satisfaction	Hennekam (2017), Zhao et al. (2018)
Knowledge resources	Niessen et al. (2012)
LMX	An (2015), Li (2015), Xu et al. (2017), Zhang (2018)
Managerial coaching	Commer et al. (2017), Raza and Ahmed (2020)
Negative affect	Porath et al. (2012)
Openness	Hennekam (2017), Hildenbrand et al. (2018)
Organizational justice	Deng (2016), Bensemmane et al. (2018)
Paradoxical leader behavior	Liu et al. (2019), Yang et al. (2021)
Perceived organizational support	Collins (2014), Abid et al. (2015), Gao D. (2017), Shen et al. (2018)
Positive affect	Porath et al. (2012)
Positive meaning	Niessen et al. (2012), Prem et al. (2017)
Proactive personality	Jiang (2017), Albi et al. (2020)
Prosocial motivation	Ghulam et al. (2018)
Psychological safety	Kark and Carmeli (2010), Xu et al. (2017), Jiang et al. (2019)
Psychological capital	Ted et al. (2013), Paterson et al. (2014), Chen et al. (2016), Levy (2016), Shen et al. (2018)
Relational resources	Niessen et al. (2012)
Self-efficacy	Gao D. (2017), Bensemmane et al. (2018), Zhu et al. (2019)
Servant leadership	Luo (2016), Walumbwa et al. (2018)
Social functioning	Zhang et al. (2020)
Supportive climate	Ted et al. (2013), Paterson et al. (2014)
Taking charge	Xu et al. (2020)
Task focus	Niessen et al. (2012), Ted et al. (2013), Paterson et al. (2014), Sia and Duari (2018)
Transformational leadership	Collins (2014), Huang (2017), Niessen et al. (2017), Dong (2018), Hildenbrand et al. (2018)
Trust	Carmeli and Spreitzer (2009), Koçak (2016), Li (2018), Xu et al. (2020)
Work control	Li (2018), Wang (2018)
Work family enrichment	Na (2017), Russo et al. (2018)
Workplace friendship	Chen et al. (2016)
Workplace civility	Ghulam et al. (2018)
Workplace incivility	Panagiotis et al. (2013)
Workplace violence	Zhao et al. (2018)

In this respect, Spreitzer et al. (2005) and Kleine et al. (2019) systematically summarized these antecedents and proposed different theoretical frameworks for the following researchers to better grasp important findings in this research field.

Specifically, Spreitzer et al. (2005) proposed a socially embedded model of thriving at work, which explained how individual learning and vitality should be integrated into social systems. They argued that employees thrive through an interaction with others in the workplace, by observation, and by communication with supervisors or colleagues. They also identified the two key predictors of thriving: “The social structural features of the focal work unit context and resources produced at work” (Spreitzer et al., 2005, p. 540). The context of the focal work unit included the social structural features of encouraging a discretion in decision-making, broad information sharing, and a climate of trust and respect. The resources produced at work included a sense of knowledge, positive meaning, positive affective resources, and relational resources. Further, they proposed three agentic work behaviors that predict thriving: task focus, exploration, and heedful relating.

Kleine et al. (2019) meta-analyzed the relationship between antecedents and thriving according to the socially embedded model of Spreitzer et al. (2005), but focused on thriving from the perspective of the characteristics of individuals and the relational resources involved. However, their model excluded the context of the work unit to be focused. Such factors included the social structural features of work demand, feedback, work autonomy, job crafting, and more. Their model also did not consider agentic work behaviors such as exploration and task focus. Furthermore, although they examined the characteristics of individuals such as core self-evaluation and proactive personality, certain personal attributes, such as self-efficacy, were excluded.

Both abovementioned frameworks are essential for us to acknowledge the indicators for employees’ thriving and fully encourage the enterprises to use corresponding strategies to motivate the employees to be more thrived. However, through a comparably comprehensive review of existing empirical studies, we find that there still exists some other important antecedents of employees’ thriving, which need further attention. Furthermore, we believe it is necessary to combine both the frameworks to figure out all possible antecedents as thoroughly as possible.

**FIGURE 1:**
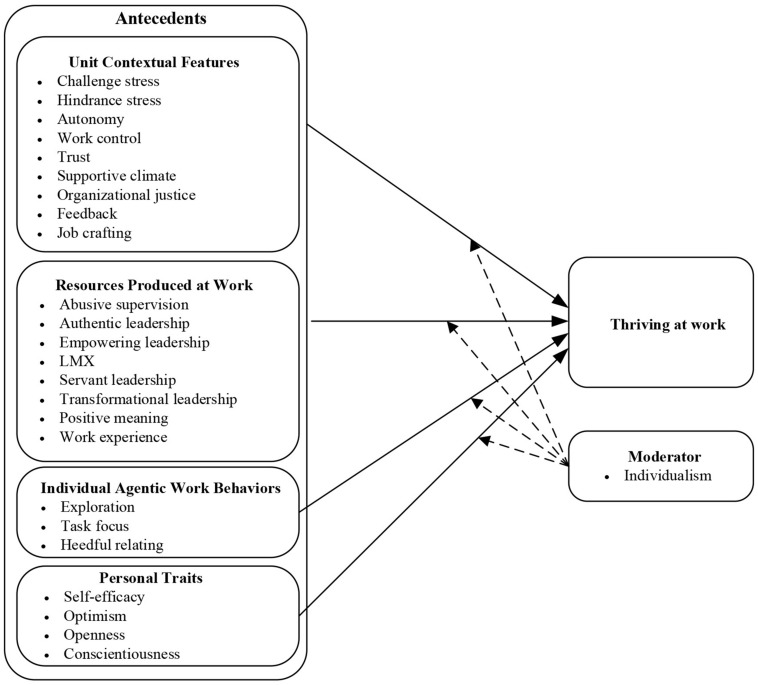
Framework of the research.

Therefore, this research extends the abovementioned basic frameworks by combining the models of Spreitzer et al. (2005) and Kleine et al. (2019) and systematically reviewing the antecedents of thriving. Considering the literary inclusion criteria of meta-analysis and some constructs, which were by Kleine et al. (2019), the study includes a comprehensive review of the effects of the contribution of unit contextual features, the resources produced at work, and the agentic work behaviors to thriving at work. Additionally, several types of personality traits, which were excluded from the previous frameworks, have been meta-analyzed. Meanwhile, this study follows a moderating effect of individualism on the links of antecedents and employees’ thriving with great interest, which has gained limited attention by existing studies. Figure 1 shows the research framework employed for this study.

## Hypothesis Development

### Unit Contextual Features

According to Spreitzer et al. (2005), reducing stressors in the workplace alone is not a sufficient encouragement for employees to thrive. Other workplace factors, such as enabling conditions, are also the crucial motivators of employees’ thriving. Therefore, unit contextual features are considered as an important indicator of thriving that should not be overlooked when examining its antecedents. Spreitzer et al. (2005) stated that unit contextual features are the characteristics of the individual work environment of an employee and include expectations, work practices, and operating procedures (Spreitzer et al., 2005, p. 541). We also argue that various workplace characteristics, work practices, and procedures may facilitate or impede a climate of information sharing, decision-making discretion, and trust. Therefore, we start with a review of the influences of various contextual characteristics on thriving.

#### Challenge Stress

It is a stressor that positively induces personal learning, growth, and accomplishment (LePine et al., 2004). Flinchbaugh et al. (2015) provided a challenge-hindrance framework, which evidenced that challenge stressors have the opposite effect on general stress, mitigate the passive effects of hindrance stressors, improve thriving, and increase employee life satisfaction. Further, their study shows that challenge appraisals indicate the magnitude of challenge stress and motivate employees to handle their daily workload effectively while actively experiencing a sense of learning. Prem et al. (2017) conducted a more detailed, diary study of the divided aspects of thriving, measuring the effects of two challenge stressors (i.e., time pressure and learning demands) in the workplace. On the two aspects of thriving, challenge stressors positively affected learning but had no impact on vitality. Generally speaking, challenge stress, which variably depends on the industry, employee’s age, tenure, and the nature of the firm, is positively associated with employee thriving (Huang, 2017). Therefore, we propose the following hypothesis:

H I-1: Challenge stress is positively related to thriving at work.

#### Hindrance Stress

In contrast to challenge stress, hindrance stress represents the pressure to perform mundane and repetitive tasks (LePine et al., 2004). According to Dollard et al. (2013), hindrance stress, measured by hindrance appraisals, is closely related to the feelings of low morale. Thus, it reduces worker satisfaction and undermines thriving (LePine et al., 2004). Additionally, when compared with challenge stress, hindrance stress discourages individual growth and its beneficial consequences as individuals use their limited resources, of time and energy, to cope with meaningless, mundane, and repetitive tasks. Subsequently, individuals perceive thriving as a strain in the workplace (Flinchbaugh et al., 2015). For example, Huang (2017) found that hindrance stress is negatively associated with thriving according to the field and online research including 542 questionnaires in East China. Therefore, we propose the following hypothesis:

H I-2: Hindrance stress is negatively related to thriving at work.

#### Autonomy

It refers to the sense of volition and control that employees experience in improving their work efficiency (Rhoades and Eisenberger, 2002). A high degree of autonomy, along with other components of intrinsic motivation, can contribute toward thriving, including vitality and learning (Geiger, 2013). Mukhaimer (2012) concluded that autonomous motivation positively affects thriving because employees feel better in a flexible work environment. According to Sia and Duari (2018), decision-making authority is critical to work satisfaction and involvement and is a key motivator for employees to thrive. Decision-making authority can enhance employee autonomy and interact with task focus, thereby motivating employees to learn and work harder. Several studies (e.g., van Scheppingen et al., 2015; Li et al., 2016) have revealed a more direct, more detailed relationship between autonomy and vitality, namely, half of thriving, whereby increased vitality stimulates the active involvement of employees, thus encouraging thriving behavior at work. Therefore, given a proper autonomy, employees experience a greater mental motivation and thriving, which effectively increases their desire to work (Gu, 2015; Xie, 2016; Gao F., 2017; Li, 2018). In light of the aforementioned studies, we propose the following hypothesis:

H I-3: Employee autonomy is positively related to thriving at work.

#### Work Control

In terms of work control, employees can manage their own work tasks and plans, granting them greater flexibility and decision-making power (Quick et al., 1990). Cheng et al. (2013) concluded that employees with more control tend to perform better and remain more positive about their work than those with less control. Therefore, the ability to control work with optimistic synergy motivates employees to confidently face the challenges of their work (Cheng et al., 2013). Additionally, strengthening the supervision and control and focusing on comparisons between work goals and outcomes improve vitality and learning processes. Employees are more likely to effectively control the quality of their work when using the quality of outcomes to set appropriate job requirements. With improved work outcomes, employees who are acknowledged for their abilities are motivated to continue working (Li, 2018). By effectively controlling work, employees can gain more resources, such as objective opinions, positive emotional, and behavioral management, which encourage employee thriving. Therefore, we propose the following hypothesis:

H I-4: Work control is positively related to thriving at work.

#### Trust

It is defined as a part of interpersonal relationships at work and plays a significant role in predicting the thriving of employees. It is the belief that another party can perform well without being forced to reach the target (Koçak, 2016). Trust can be divided into three aspects: a management team, a supervisor, and colleagues. Trust in a management team is developed when an employee feels supported and shows that he/she relies on the decisions of the organization. The relationship between employees and their supervisors and/or colleagues also emphasizes trust on a smaller scale (Koçak, 2016). High-quality relationships are beneficial in motivating the employees to be more active at work and when interacting with supervisors and colleagues, and ultimately thrive more (Li, 2018). Additionally, employee trust in leaders equips leaders with power and authority for management, and trust in employers ensures that employers are more capable of supervision and learn from colleagues. With the proper guidance of supervisors and colleagues, employees are more willing to thrive in a voluntary manner (Jaiswal and Dhar, 2017). Through collective trust-based interactions, employees enjoy high-quality social bonds and a higher level of thriving at work. Carmeli et al. (2009) explored the relationship between positive work relationships and vitality in an alternative explanatory path. With trust, building high-quality relationships encourages employees to have the confidence to actively express their personal opinions. It also enables employees to respect the requirements of their work. Therefore, we propose the following hypothesis:

H I-5: Trust is positively related to thriving at work.

#### Supportive Climate

The workplace environment can positively or negatively affect employees (Paterson et al., 2014). In a supportive and friendly environment, supervisors tend to pay attention to their subordinates and express concern in many aspects, including work performance and individual development. They also tend to provide a variety of resources to help with employee tasks. These behaviors facilitate the work of an employee and encourage them to work agentically, which leads them to thrive (Paterson et al., 2014). Additionally, the social support provided by supervisors and colleagues can be helpful in supporting employees to feel secure in their jobs. In short, under a supportive climate, unconfident or confused employees are motivated by suggestions for improvement, increasing their likelihood of thriving well for a longer period of time (Cheng et al., 2013). Zhu et al. (2019) discovered the positive effect of a supportive environment among a particular group of workers, namely, people with disabilities. They also suggested that a climate of team learning can reduce the negative impact of disability and ultimately enhance the thriving of people with disabilities. Therefore, we propose the following hypothesis:

H I-6: A supportive workplace climate is positively related to thriving at work.

#### Organizational Justice

It is perceived as the opinion of an employee on workplace equity (Colquitt et al., 2001). Bensemmane et al. (2018) argued that, despite an individual variation, the overall average sense of justice across a team contributes to the collective thriving of team members. Mushtaq et al. (2017) uncovered the mechanisms underlying the influence of justice on employee thriving and suggested that employees with a higher sense of organizational justice show greater confidence in the assessment and reward system of an organization. Given this belief, employees concentrate on their work and pursue performance improvement, which translates to a higher degree of thriving. Working in an organization that emphasizes the improvement of three components of justice, including procedural, interactional, and distributive justice, drives the motivation for employees to thrive (Deng, 2016). Thus, we propose the following hypothesis:

H I-7: Organizational justice is positively related to thriving at work.

#### Feedback

It is a way for supervisors to provide information to subordinates according to the outcomes of their work (Maurer et al., 2002). Useful feedback not only provides an incentive for employees to learn new skills and ideas but also potentially transforms the workplace to an open and a transparent system, significantly increasing employee thriving (Xie, 2016). In terms of an integrative model of human growth in the workplace, Gao F. (2017) concluded that supervisory developmental feedback positively affects the basic psychological needs of employees and further influences their thriving. Supervisors use verbal encouragement and express concern to build relationships with their subordinates. This effective interaction strengthens employees’ sense of belonging to the organization and guides them on how to improve their work, which ultimately stimulates thriving (Wang et al., 2017). Lee et al. (2015) stated that feedback-seeking behavior in a team feedback environment can also significantly improve the work performance and attitude of employees, leading to increased thriving as employees become clear about their work abilities and responsibilities. Therefore, we propose the following hypothesis:

H I-8: Work feedback is positively related to thriving at work.

#### Job Crafting

It refers to the behavior of an employee in modifying and adjusting the content of a job to improve performance; a process that clarifies and confirms their job requirements and their own beliefs (Wrzesniewski and Dutton, 2001). This leads to the conclusion that employees with positive and enthusiastic attitudes toward work ultimately experience thriving (Li, 2015). Employees who craft their jobs strengthen their awareness of their duties and abilities. After proper positioning, they use organizational resources more efficiently and feel motivated to learn on their jobs (Wang, 2018). Additionally, job crafting enables employees to take on challenges, advance their knowledge, and lay the foundation for their personal growth. Through this process, the advantages of concentration and progress at work motivate employees with crafted jobs to thrive at a higher level (Han, 2017). Therefore, we propose the following hypothesis:

H I-9: Job crafting is positively related to thriving at work.

### Resources Produced at Work

There are other factors, in addition to the influence of the different unit contextual features on employee learning and vitality, which influence employee thriving, including the resources produced at work. In the socially embedded model of thriving of Spreitzer et al.’s (2005), various types of resources can facilitate employee’s thriving, such as knowledge resources, a positive meaning, and relational resources. In this section, according to the existing empirical research, we perform a meta-analysis to examine the effects of various types on leadership, defined as a type of relational resources, in addition to the positive meaning and knowledge resources (work experience).

#### Abusive Supervision

It is defined as a “subordinate’s perception of the extent to which supervisors engage in a sustained display of hostile, verbal, and non-verbal behaviors, excluding physical contact” (Zhang and Bednall, 2016, p. 455). According to the theory of affective events, researchers argue that abusive supervision is negatively associated with thriving (Luo, 2016). Employees perceive abusive supervision as a negative affective event that undermines the climate of trust and respect; a climate that ensures employee safety and encourages them to take risks and explore. Without this, employees can feel untrusted or disrespected and can lose their energy and enthusiasm for learning at work. Therefore, we propose the following hypothesis:

H II-1: Abusive supervision is negatively related to thriving at work.

#### Authentic Leadership

It is a specific leadership style that focuses on the self-awareness and psychological capital of both workplace leaders and followers (Gardner et al., 2005). Under this kind of leadership, leaders driven by their own values and beliefs provide more support to their staff and pursue an equal work environment (Gardner et al., 2005). Therefore, a positive relationship may exist between authentic leadership and employee thriving. Xu et al. (2017) emphasize this finding by highlighting a positive association between authentic leadership and thriving, especially through the meditating variable of leader–member exchange (LMX). LMX improves communication and interaction from the team level to an individual level, so that authentic leaders can influence their followers, in supervisory interactions, by increasing an inherent motivation for self-determination (Ryan and Deci, 2000). Therefore, we propose the following hypothesis:

H II-2: Authentic leadership is positively related to thriving at work.

#### Empowering Leadership

Unlike authentic leadership, empowering leadership emphasizes delegation to subordinates to improve their psychological experience at work (Li et al., 2016). Delegation is a process of power-sharing, whereby employees have flexibility in decision-making, setting goals, or completing tasks. Simultaneously, they are more likely to feel motivated to learn on their own as they realize more responsibility and confidence from their leader (Ali et al., 2018). Empowered employees tend to shoulder more responsibilities than originally required (Kabat-Farr and Cortina, 2017). In the process, they are vitalized and become willing to learn to improve their work performance. Along with this opinion, Li et al. (2016) also proposed that empowering leadership engenders thriving. Specifically, this leadership style contributes to a greater tendency for employees to thrive, by encouraging them to participate in decision-making processes and providing many supportive resources. As a result, employees are highly willing to learn, stay vitalized, and feel more secure and responsible at work (Han, 2017). Therefore, we propose the following hypothesis:

H II-3: Empowering leadership is positively related to thriving at work.

#### Leader–Member Exchange

It refers to a particular relationship between leaders and their subordinates built *via* exchanges at work. Atwater and Carmeli (2009) concluded that the higher the quality of LMX, the more employees engage, and the more willing they are to be active and passionate at work because they feel trusted and respected. In short, harmonious LMX leads to employee thriving and improves work outcomes (Li, 2015; Xu et al., 2017). Zhang et al., (2018) drew a similar conclusion that LMX facilitates emotional bonding between leaders and their subordinates beyond the simpler relationship between the colleagues. This bond enhances the efficiency of employees, encourages engagement, brings vitality, and promotes learning. Thus, high-quality LMX between leaders and employees beneficially facilitates their work efficiency and enriches their job content (Atwater and Carmeli, 2009; Zhang et al., 2018). Therefore, we propose the following hypothesis:

H II-4: LMX is positively related to thriving at work.

#### Servant Leadership

Previous studies have suggested that there is a strong relationship between servant leadership and thriving at work (e.g., Jaiswal and Dhar, 2017; Walumbwa et al., 2018). Servant leadership is a positive leadership approach, focusing on serving colleagues and supporting them *via* necessary work resources and compassion. This empowers employees to take on challenges and be confident in continuously improving themselves (Walumbwa et al., 2018). Furthermore, servant leadership can lead to employee trust in leadership and enhance creativity in thriving (Jaiswal and Dhar, 2017). Luo (2016) revealed a positive correlation between servant leadership and thriving by investigating the effect of aggressive mistreatment by customers on employee thriving. Therefore, we propose the following hypothesis:

H II-5: Servant leadership is positively related to thriving at work.

#### Transformational Leadership

It exists when leaders actively stimulate employee motivation and help them achieve higher goals (Collins, 2014). The relationship between thriving and transformational leadership has recently attracted attention, and current empirical evidence has proven to be very positive (Dong, 2018). Transformational leadership enables employees to experience beneficial encouragement, happiness, and openness at work while encouraging them to feel more autonomous in a respectful and supportive environment. This collectively leads to the thriving of individual employees (Hildenbrand et al., 2018). Furthermore, this leadership style motivates employees to learn and increase vitality *via* social capital. Niessen et al. (2017) shed light on the relationship between the perceived transformational leadership and the thriving of teachers. Although they did not conclude a direct relationship between them and revealed that employees felt increased levels of thriving under strong transformational leadership, given that they were less emotionally exhausted. Therefore, we propose the following hypothesis:

H II-6: Transformational leadership is positively related to thriving at work.

In addition to various types of leadership, also known as relational resources (Spreitzer et al., 2005), two types of resources were meta-reviewed: positive meaning and knowledge resources (work experience).

#### Positive Meaning

It involves both the purpose and significance of work. It comes from the value of work and the creation of worth *via* interacting with colleagues at work (Spreitzer et al., 2005). Positive meaning at work aligns the expectations of employees to each other and also helps them to understand the goals of the organization. It enables them to understand their work and its significance and engenders thriving at work. Niessen et al. (2012) conducted a diary study and showed a positive relationship between personal variability during workdays and positive meaning and thriving. Their study provides evidence for a number of hypotheses for the socially embedded model of thriving proposed by Spreitzer et al. (2005), and expands the literature by focusing on identifying personal differences in positive meaning in a single day alone rather than concentrating on personal growth- and work-related outcomes. Individuals who experience a stronger sense of positive meaning at work will feel a sense of thriving on the same day. Therefore, we propose the following hypothesis:

H II-7: Positive meaning is positively related to thriving at work.

#### Work Experience

In the model of Spreitzer et al. (2005), knowledge resources are known as the important resources generated at work that engender employee thriving. We argue that work experience is a knowledge resource because it is generated at work and enables employees to understand their work; in other words, it is related to the knowledge required and how to obtain the relevant information to get the job done (Moreland and Argote, 2003). Therefore, we expect that work experience serves as a knowledge resource for employees to learn about how to acquire the knowledge, information, and skills needed to work and promote thriving. Therefore, we propose the following hypothesis:

H II-8: Work experience is positively related to thriving at work.

### Individual Agentic Work Behaviors

Also known as the engine of thriving (Spreitzer et al., 2005), agentic work behaviors include exploration, task focus, and heedful relating, and have significant effects on the thriving of an individual. This section focuses on the effects of these three behaviors on thriving.

#### Exploration

It is a phenomenon in which individuals seek new ways of working, *via* experimentation, discovery, innovation, and risk-taking (Spreitzer et al., 2005). Previous studies have evidenced the positive relationship between exploration and thriving (Button et al., 1996). Spreitzer et al. (2005) posited that, when employees experienced explore at work, they were willing to acquire new knowledge to resolve problems, therefore positively related to the thriving of an employee. Exploration behavior also enhances employee vitality; Kleine et al. (2019) stated that exploration is a direct outcome of thriving as the activity is associated with the immediate experience of both vitality and learning. Sia and Duari (2018) evidenced the positive result of thriving by finding that work exploration stimulates employees to produce surprising ideas and strategies. Therefore, we propose the following hypothesis.

H III-1: Exploration is positively related to thriving at work.

#### Task Focus

Although the importance of heedful relating has attracted much attention, it is also essential, in investigating thriving at work, to analyze individual work task characteristics such as task focus. Task focus refers to the dedication and concentration required by an employee to achieve the objectives of a job (Brown et al., 2005). Niessen et al. (2012) concluded that task focus is a key agentic work behavior that plays an important role in increasing employee thriving. Specifically, highly dedicated employees focus on completing tasks and finding the best possible solutions, resulting in a higher likelihood of success and molding them into vital, active-learning employees. Employees who are highly task-focused feel a sense of accomplishment at work and actively acquire, previously unknown, integral job skills; thereby increasing thriving (Paterson et al., 2014). Furthermore, Paterson et al. (2014) found that, to thrive in a variety of job roles, employees adopt various behaviors depending on the scenario. People tend to be attracted to work by the tasks involved in the job, ergo, and are inclined to feel more vitality from these tasks and actively learn what is necessary to complete them (Sia and Duari, 2018). Therefore, we propose the following hypothesis.

H III-2: Task focus is positively related to thriving at work.

#### Heedful Relating

It refers to cooperative and mutually supportive interactions between the workers (Paterson et al., 2014). Many people work in advanced teamwork-based organizations, where collaboration and communication with teammates significantly affect employee thriving. In this case, the focus is on the interactions within the group and the achievements of the entire team. More effective and high-quality cooperation between the employees, that is, more heedful relating, engenders higher levels of thriving (Paterson et al., 2014). Heedful relations between colleagues are important for employees to fully realize the functions of the organizational structure and the ultimate goals of the organization. Through intensive and high-quality interactions, employees tend to actively learn from their colleagues and play responsible, larger roles in the team, ultimately increasing their levels of thriving (Sia and Duari, 2018). Abid et al. (2016) conducted an empirical study in support of the positive effects of heedful relations and posited that heedful relating motivates positive employee behavior, which is consistent with previous arguments. Therefore, we propose the following hypothesis:

H III-3: Heedful relating at work is positively related to thriving at work.

### Personality Traits

In addition to the above antecedents in the model of thriving by Spreitzer et al. (2005) and Kleine et al. (2019) meta-analyzed several types of personality traits such as core self-evaluation and proactive personality. For this study, we meta-analyzed the relationship between the other four types of personality traits and thriving at work to complement the models of Spreitzer et al. (2005) and Kleine et al. (2019).

#### Self-Efficacy

It implies having an optimistic attitude toward difficulties and hurdles (Geiger, 2013). The relationship between self-efficacy and thriving has recently attracted attention as evidencing significantly positive effects on thriving, regardless of employee group (Geiger, 2013; Lee et al., 2015; Chen et al., 2016; Levy, 2016; Bensemmane et al., 2018; Zhu et al., 2019). Building self-efficacy, as a part of intrinsic mindfulness, increases the likelihood of employees thriving regardless of workplace characteristics, including the organizational climate (Geiger, 2013). Moreover, employees with high self-efficacy are ready to face the challenges at work and are confident enough to complete them. Thus, as self-efficacy accumulates, employees are more likely to feel that they are thriving regardless of the work environment (Levy, 2016). Bensemmane et al. (2018) discovered this relationship in the context of a team and proposed that self-efficacy is integral to understanding the importance and effectiveness of personal resources. Therefore, we propose the following hypothesis.

H IV-1: Self-efficacy is positively related to thriving at work.

#### Optimism

It is a personality trait that refers to the ability to positively associate events with outcomes (Luthans et al., 2007). Similar to the analytical path of self-efficacy, Levy (2016) agreed that optimism encourages employees to focus on their work, which increases the likelihood of meeting the job requirements and completing difficult tasks, thereby contributing to their experience of thriving. Xie (2015) also proposed that optimism is one of the four components of psychological capital. Employees are believed to thrive more from frustration when they use psychological capital, such as the patience and courage offered by optimism, to manage challenges or to face unfair treatment. Furthermore, optimistic employees accept challenges, with confidence and a positive attitude, making them less likely to be influenced by anxiety or anger. Thus, they gain more positive outcomes such as thriving (Cheng et al., 2013). Therefore, we propose the following hypothesis.

H IV-2: Optimism is positively related to thriving at work.

#### Openness

Employees who are open tend to show positive emotions and also tend to be active and energetic at work. Additionally, open-minded employees are modest and inherently learn from their surroundings (Hennekam, 2017) and, therefore, experience higher levels of thriving. The interaction between being open to experience and transformational leadership also affects employee thriving (Hildenbrand et al., 2018). In the context of leadership, people who are open are able to cope with opposing voices and to avoid serious embarrassment at work, thus supporting them to thrive. By analyzing the underlying mechanisms, we propose the following hypothesis.

H IV-3: Openness is positively related to thriving at work.

#### Conscientiousness

It describes the ability of an individual to maintain self-discipline and continued motivation to achieve goals (Costa et al., 1991), and has been found to positively influence employee thriving. Conscientious employees tend to behave well and work hard toward their goals as they are familiar with their work and job contents (Hennekam, 2017). Simultaneously, this ability leads to outperformance and provides a sense of accomplishment. Therefore, conscientious employees tend to thrive easily (Hennekam, 2017). Conscientiousness also expresses a sense of responsibility that helps employees motivate themselves to thrive, and the employees tend to work more eagerly and broadly, laying a foundation for the emergence of thriving (Xie, 2015). Therefore, we propose the following hypothesis.

H IV-4: Conscientiousness is positively related to thriving at work.

#### The Moderating Effects of National Culture

Most research on management specifies national culture as an import contingent factor that influences the relationships between different variables (Rattrie et al., 2020). For example, it serves as an important contextual factor that will influence the relationships between some work-related antecedents and outcomes (e.g., Choi et al., 2015, personality traits and organizational commitment). The dimensions of national culture were also regarded as influential factors that determine the responses of individuals toward work conditions and subsequently the associated results of the work (Liu et al., 2007; Taras et al., 2010, 2011). According to his research on national culture, Hofstede (1984) identified individualism as an important characteristic of each country and revealed the contrast in the scores of individualism between Eastern, primarily Asian countries, and Western countries. Compared to China with a score of being 20, the United States had a much higher score of 91 (Hofstede, 2001). Several studies are consistent with this result, including the regulatory focus theory of Higgins (1997) as well as the other relevant disparities between the scores.

Among the dimensions of national culture, individualism/collectivism dimension was considered as one of the important factors that cause the deviation of the reactions of individuals toward different job characteristics (e.g., Spector et al., 2007) and that of the different levels of their commitment to the organizations (Parkes et al., 2001). Individuals with an interdependent self-construal, in low-level individualistic countries, actively respond to information about accepting, or avoiding, responsibility for their surroundings; whereas, those with an independent self-construal, in high-level individualistic countries, tend to react to information in terms of personal goals and objectives (Lee et al., 2000; Elliot et al., 2001; Xu et al., 2018). Citizens living in high-scoring, individualistic countries tend to prioritize their interests, and set themselves apart from others. Therefore, the relationship between antecedents and thriving varies depending on the levels of individualistic culture.

According to the socially embedded model of thriving, the thriving of an individual is not only self-driven but also occurs through his/her interactions with others (Brown and Duguid, 1991; Wenger, 1998). Low-level individualistic countries emphasize the importance of collective interests in a group level and encourage individuals to support each other and maintain harmonious relationships. Individuals in low-level individualistic countries “are integrated into strong, cohesive in-groups, which throughout people’s lifetime continue to protect them in exchange for unquestioning loyalty” (Hofstede, 2001, p. 225). Ronen and Mikulincer (2009) also stated that individuals in low-level individualistic cultures were more sensitive to the fulfillment of group goals, the completion of teamwork, and the improvement engagement in a group level (Rattrie et al., 2020). This necessary interaction brought by the teamwork among individuals, *via* culture, is more likely to facilitate employee thriving in benign work environments, with positive work unit contextual features and resources. Therefore, we propose the following hypothesis.

H V: The relationship between antecedents and employee thriving is stronger in the countries with low-level of individualism than in the countries with high-level of individualism.

## Methods

### Search Strategy

To identify the empirical studies on thriving at work for use in the meta-analysis, we followed the search procedures in other meta-analysis (e.g., Zhang et al., 2017) and employed the following strategy to locate appropriate articles. Firstly, we conducted a computer-based search in the databases, including Web of Science (SSCI), EBSCO, ABI/INFORM, ERIC, PsycINFO, Google Scholar, and Scopus by filtering the date prior to 2019, and using the keywords “work” and “thriving” or two dimensions of thriving, namely “vitality” and “learning.” We also search for the articles containing the term “工作繁荣”和“工作旺盛感” in China National Knowledge Infrastructure (CNKI), which is the most commonly used database for searching Chinese academic publications. Secondly, we manually searched for recent empirical articles that address thriving *via* qualitative and/or quantitative review (Kleine et al., 2019). For unpublished studies, we took four search approaches into dissertations, reports, book chapters, working papers, and conference papers. Firstly, dissertations were searched for in the ProQuest databases. Secondly, book chapters, working papers, and conference papers were searched for on Scopus and Web of Science. Finally, we distributed the information about our meta-analysis on the service lists of the Human Resources and Organizational Behavior Divisions at the Academy of Management conference and extracted additional working papers on thriving at work.

### Inclusion and Exclusion Criteria

The following procedure was used to identify the eligible studies that could be used for meta-analysis. Firstly, empirical studies must include the variable to be focused on, that is thriving at work, and must be categorized within the discipline of management. Secondly, at least one of the antecedents for thriving, from the current model, must be included in the study. Thirdly, thriving must be measured empirically with the reported correlations between thriving at work and its antecedents. Finally, 67 current studies (*N* = 28,097) were included in the final sample.

### Coding Procedures

Prior to the coding process, two researchers of this study developed a coding scheme according to Krippendorff (2013) to guarantee consistency between different coders. Specifically, to decrease the inconsistency of the concepts among the antecedents, which are similar but termed in different forms (e.g., challenge stress/challenging stress), two coders had a discussion with each other to ensure the consistency of a related term in the coding scheme. In addition, concepts such as “challenge demand” in some papers were coded as challenge stress as these two constructs fully resemble each other. In this study, as shown in Table 2, similar constructs are used to describe specific antecedents. Next, according to this scheme, two coders independently coded the data from the selected empirical studies. The coded information for each study included: (1) the correlations between thriving and its antecedents, (2) the sample size, (3) Cronbach’s alpha of thriving and its antecedents, and (4) the moderators, which specify the countries, wherein each study was conducted. After completing their task, each coder independently checked the coding sheets of each other, and in case of existence of any inconsistencies, the disagreement was discussed and addressed. The inter-rater reliability of the two coders was high (Cohen’s kappa = 0.88).

**TABLE 2:** Summary of similar constructs used to identify specific antecedents.

Antecedents	Similar constructs
**Unit contextual features**	
Challenge stress	Challenge stress
	Challenge demand
	Challenge appraisal
Hindrance stress	Hindrance stress
	Hindrance demand
	Hindrance appraisal
	Role ambiguity
	Role overload
	Time pressure
Autonomy	Autonomy
	Work autonomy
	Autonomy orientation
	Decision-making authority
	Autonomous motivation
	Flexibility-autonomy
	Need for autonomy
Work control	Work control
	Job control
	Sense of work control
Trust	Trust
	Trust in leader
	Trust in Colleagues
	Trust in Supervisor
	Interpersonal trust
Supportive climate	Supportive climate
	Supportive supervising style
	Team-learning climate
Organizational justice	Organizational justice
	Fairness Perception
	Average transient overall team justice
Feedback	Supervisor feedback
	Feedback-seeking behavior
	Supervisor developmental feedback
Job crafting	Job crafting
**Resource produced at work**	
Abusive supervision	Abusive supervision
	Abusive management
	Workplace violence
Authentic leadership	Authentic leadership
Empowering leadership	Empowering leadership
	Empowerment
LMX	Leader–member exchange
	LMX
	LMX quality
	Supervisor-subordinate relationship
	Leader relational behaviors
	Social exchange
Servant leadership	Servant leadership
Transformational leadership	Transformational leadership
	Perceived transformational leadership
Positive meaning	Positive meaning
Work experience	Experience
	Work experience
	Number of organizations one has worked in
**Personal traits**	
Self-efficacy	Self-efficacy
	Average transient self-efficacy
	Innovation self efficacy
	Job self-efficacy
Optimism	Optimism
Openness	Openness
	Openness to experience
	External work contacts
Conscientiousness	Conscientiousness
**Individual agentic work behaviors**	
Exploration	Exploration
	Active exploration
Task focus	Task focus
	Concentration
Heedful relating	Heedful relating

### Sample Information

Most of the empirical studies were conducted in China and the United States, which account for 50 and 7.81%, respectively. The samples included male and female participants with the percentage of men being greater than 50% accounting for 61.40%, and women being greater than 50% accounting for 38.6%. Most of the research subjects were in the range of 30–40 years old. Details about each sample are shown in Table 3.

**TABLE 3:** Summary of sample information.

Category	Percentages
**Gender (% of male)**	
<=50%	61.40%
>50%	38.60%
**Average age**	
>20 and ≤30	24.14%
>30 and ≤40	44.83%
>40 and ≤50	27.59%
>50	3.45%
**Country**	
Austria (Central Europe)	1.56%
Belgium (Central Europe)	1.56%
Canada	4.69%
China	50.00%
Finland	1.56%
France	1.56%
Germany (Central Europe)	4.69%
India	3.13%
Indonesia	1.56%
Israel	6.25%
Korea	1.56%
Netherlands	3.13%
Pakistan	3.13%
South Africa	3.13%
Taiwan	1.56%
Turkey	1.56%
United States	7.81%
Worldwide	1.56%

### Publication Bias

Two methods were used to expose the publication bias in the analysis. Firstly, we used the fail-safe N of Rosenthal (1979) as a measure of the number of existing, unpublished studies needed to transform a significant population effect size estimate into a non-significant result. Table 4 shows the results of all the fail-safe Ns for each bivariate relationship in this study. On an average, our sample had a fail-safe *N* of 893.5, suggesting that 893.5, or more, unpublished studies should be included in the analysis to reduce the size of the population effect to a non-significant level. Secondly, after deriving the number of unreleased publications needed to change the result, we calculated if it was actually influenced by a publication bias. In Begg and Mazumdar (1994), the Kendall rank correlation coefficient was used to test the effect on the significance of the results. As shown in Table 4, the non-significant Kendall rank correlation coefficient indicates that all relationships are independent of publication bias. These results show that most of the tested correlations (24 out of 24, i.e., 100%) are not influenced by publication bias.

**TABLE 4:** The results of publication bias test.

Antecedents	*k*	*N*	Classic Fail-safe *N*	Kendall’s Tau	*P*
***Unit contextual features***					
Challenge stress	6	2,580	669	−0.27	0.452
Hindrance stress	9	5,610	435	0	1
Autonomy	11	3,883	3679	−0.121	0.583
Work control	7	3,161	680	0.19	0.548
Trust	8	2,418	882	−0.07	0.851
Supportive climate	5	2,176	271	−0.67	0.296
Organizational justice	3	1,013	553	−0.33	0.601
Feedback	5	2,423	1931	0	1
Job crafting	3	803	534	−0.33	0.601
***Resources produced at work***					
Abusive supervision	3	1,602	55	−0.67	0.296
Authentic leadership	5	1,819	54	0.33	0.601
Empowering leadership	6	2,364	167	0	1
LMX	9	2,871	2636	−0.25	0.348
Servant leadership	5	2,028	271	−0.67	0.296
Transformational leadership	6	2,121	882	−0.07	0.851
Positive meaning	4	631	320	−0.1	0.81
Work experience	6	2,537	8	0.5	0.22
***Individual agentic work behaviors***					
Exploration	3	509	536	−0.8333	0.089
Task focus	6	1,751	1373	−0.13	0.707
Heedful relating	12	3,149	121	0.1	0.806
***Personality traits***					
Self-efficacy	9	3,606	1580	0.107	0.711
Optimism	4	1,856	1109	0.17	0.734
Openness	3	1,470	1109	0.17	0.734
Conscientiousness	5	2,702	599	0.1	0.807

*k, the number of independent effect sizes included in each analysis; *N*, the number of participants in each analysis; Classic fail-safe *N*, the number of unpublished studies it will take to raise the *p-*value to an insignificant level; Kendall’s Tau, Kendall rank correlation coefficient; *p*, the *p-*value for Kendall rank correlation coefficient.*

### Analysis

A random effects model was implemented to test hypotheses I–IV, which were proposed to describe the relationships between thriving and its outcomes. In Hunter and Schmidt (2004), an approach to psychometric meta-analysis was used as it considers, in the field of organizational behavior, the influence of artifacts such as measurement error—a common issue in psychometric empirical research. Regarding the mean effect, we report the independent effect size (*k*), sample size (*N*), and weighted mean correlation (*r*). We also report mean true-score correlation ($\bar{\rho }$); and observe SDs of corrected correlations [SD_(r_c)], residual SDs of ρ (SD_ρ), the 95% CI for the main effect, and the variability of corrected effect size estimates investigated by calculating 80% credibility intervals.

As mentioned and according to the previous research on cross-cultural meta-analysis, national culture has various moderating effects on the results of this study (e.g., Liu et al., 2016). The website of Geert Hofstede^1^ displays the national scores, calculated by representative country samples, from the World Values Survey. These scores were assigned in an ascending order from 0 to 100 to accurately measure the individualism culture dimension. These scores were then matched according to the samples in our meta-analysis. The final step was to conduct a meta-regression (Borenstein et al., 2009) to test the significance of the moderating effect of long-term orientation.

## Results

### The Influence of Work Unit Contextual Features on Thriving

From H I-1 and H I-2, it is inferred that unit contextual features such as hindrance stress are negatively related to thriving at work. In Contrast, challenge stress is positively related to thriving at work. As shown in Table 5, challenge stress ($\bar{\rho }\,$ = 0.46; H I-1) is positively associated with thriving, and hindrance stress ($\bar{\rho }\,$ = − 0.19; H I-2) is negatively associated with thriving.

**TABLE 5:** Meta-analysis of relationships between thriving at work and its antecedents.

Antecedents	*k*	*N*	$\bar{r}$	SD_r_	SD_res_	$\bar{\rho }$	SD_rc_	SD_p_	95% CI	80% CR
***Unit contextual features***										
Challenge stress	6	2,580	0.39	0.18	0.18	0.46	0.19	0.19	(0.30,0.61)	(0.22, 0.70)
Hindrance stress	9	5,610	−0.17	0.11	0.10	−0.19	0.12	0.11	(−0.27, −0.11)	(−0.33, -0.04)
Autonomy	11	3,883	0.42	0.18	0.17	0.49	0.20	0.19	(0.38, 0.61)	(0.25, 0.74)
Work control	7	3,161	0.21	0.29	0.29	0.26	0.34	0.34	(0.00, 0.51)	(−0.18, 0.69)
Trust	8	2,418	0.35	0.16	0.15	0.49	0.20	0.19	(0.35, 0.62)	(0.25, 0.72)
Supportive climate	5	2,176	0.35	0.15	0.16	0.40	0.17	0.16	(0.26, 0.54)	(0.19, 0.60)
Organizational justice	3	1,013	0.59	0.16	0.15	0.67	0.16	0.15	(0.49, 0.84)	(0.47, 0.86)
Feedback	5	2,423	0.53	0.12	0.12	0.62	0.14	0.14	(0.50, 0.75)	(0.45, 0.80)
Job crafting	3	803	0.51	0.06	0.03	0.61	0.09	0.07	(0.51, 0.71)	(0.52, 0.70)
***Resources produced in doing of work***										
Abusive supervision	3	1,602	-0.17	0.06	0.05	-0.21	0.07	0.05	(−0.29, -0.13)	(−0.27, -0.15)
Authentic leadership	5	1,819	0.32	0.19	0.18	0.38	0.22	0.21	(0.19, 0.57)	(0.11, 0.65)
Empowering Leadership	6	2,364	0.39	0.15	0.14	0.47	0.16	0.15	(0.35, 0.60)	(0.28, 0.66)
LMX	9	2,871	0.49	0.13	0.12	0.59	0.15	0.14	(0.50, 0.69)	(0.42, 0.77)
Servant leadership	5	2,028	0.39	0.10	0.09	0.49	0.12	0.10	(0.40, 0.59)	(0.36, 0.62)
Transformational leadership	6	2,121	0.42	0.19	0.19	0.46	0.21	0.21	(0.29, 0.64)	(0.20, 0.73)
Positive meaning	4	631	0.46	0.07	0.04	0.53	0.09	0.06	(0.44, 0.62)	(0.45, 0.60)
Work experience	6	2,537	0.05	0.09	0.08	0.05	0.11	0.10	(−0.03, 0.13)	(−0.07, 0.18)
**Individual agentic work behaviors**										
Exploration	3	509	0.55	0.04	0.00	0.66	0.06	0.00	(0.59, 0.73)	(0.66, 0.66)
Task focus	6	1,751	0.54	0.12	0.11	0.63	0.14	0.13	(0.52, 0.74)	(0.47, 0.80)
Heedful relating	12	3,149	0.42	0.13	0.11	0.52	0.13	0.12	(0.45, 0.59)	(0.37, 0.67)
**Personality traits**										
Self-efficacy	9	3,606	0.41	0.16	0.15	0.48	0.16	0.15	(0.38, 0.58)	(0.28,0.68)
Optimism	4	1,856	0.54	0.03	0.01	0.65	0.05	0.03	(0.60, 0.70)	(0.62, 0.69)
Openness	3	1,470	0.06	0.07	0.06	0.07	0.08	0.06	(−0.02, 0.16)	(−0.01, 0.15)
Conscientiousness	5	2,702	0.35	0.14	0.13	0.40	0.14	0.14	(0.28, 0.53)	(0.23, 0.58)

*k, number of studies contributing to meta-analysis; *N*, total sample size; $\bar{r}$, mean observed correlation; *SD*_*r*_, observed standard deviation of *r*; *SD*_*res*_, residual standard deviation of *r*; $\bar{\rho }$, mean true-score correlation; *SD*_*rc*_, observed standard deviation of corrected correlations (*r*_*c*_); *SD*_*p*_, residual standard deviation of *P*; CI, confidence interval around $\bar{\rho }$; CR, credibility interval around $\bar{\rho }$. Correlations corrected individually.*

From H I-3 and H I-4, it is inferred that that autonomy ($\bar{\rho }=0.49$; H I-3) and work control ($\bar{\rho }\,$ = 0.26; H I-4) are moderately and positively related to thriving at work. All 95% CIs exclude zero. Therefore, H I-3 and H I-4 are supported.

From the hypotheses H I-5 to H I-9, it is inferred that trust, supportive climate, organizational justice, feedback, and job crafting is positively related to thriving. As shown in Table 5, trust $\bar{\rho }\,$ = (0.49; H I-5), supportive climate ($\bar{\rho }\,$ = 0.40; H I-6), organizational justice ($\bar{\rho }$ = 0.67; H I-7), feedback ($\bar{\rho }\,$ = 0.62; H I-8), and job crafting ($\bar{\rho }\,$ = 0.61; H I-9) have moderate to strong, positive effects on thriving as all 95% CIs exclude zero. Therefore, H I-5 to H I-9 are supported.

### The Influence of Resources Produced at Work, on Thriving

H II-1 proposes that negative leadership such as abusive supervision is negatively related to thriving. As shown in Table 5, abusive supervision is negatively associated with thriving ($\bar{\rho }\,$ = − 0.21, H II-1). H II-1 is supported because the 95% CIs of these estimated relationships exclude zero. H II-2 to H II-6 propose that positive leadership, including authentic, empowered, servant, and transformational leaderships, as well as LMX and trust, is positively related to thriving. As shown in Table 5, authentic leadership ($\bar{\rho }\,$ = 0.38; H II-2), empowered leadership ($\bar{\rho }\,$ = 0.47; H II-3), LMX ($\bar{\rho }\,$ = 0.59; H II-4), servant leadership $\bar{\rho }\,$ = 0.49; H II-5), and transformational leadership ($\bar{\rho }\,$ = 0.46; H II-6) are moderately and positively correlated with thriving as all 95% CIs exclude zero. Therefore, H II-2 to H II-6 are supported.

H II-7 proposes that positive meaning is positively related to thriving. As shown in Table 5, positive meaning ($\bar{\rho }\,$ = 0.53) is moderately and positively correlated with thriving as all 95% CIs exclude zero. H II-8 proposes that work experience, as one type of knowledge resources, positively influences thriving. The results in Table 5 show that work experience ($\bar{\rho }\,$ = 0.05) is not related to thriving as the 95% CIs include zero. Therefore, H II-7 and H II-8 are partially supported.

### The Influence of Individual Agentic Work Behaviors on Thriving

H III-1 to H III-3 propose that individual agentic work behaviors are positively related to thriving. The results in Table 5 indicate that exploration ($\bar{\rho }\,$ = 0.66; H III-1), task focus ($\bar{\rho }\,$ = 0.63; H III-2), and heedful relating ($\bar{\rho }\,$ = 0.52; H III-3) have moderate to strong, positive correlations with thriving as all 95% CIs exclude zero. Therefore, H III-1 to H III-3 are supported.

### The Influence of Personality Traits on Thriving

H IV-1 and H IV-2 propose that positive personality traits, including self-efficacy, optimism, openness, and conscientiousness, are positively related to thriving. As shown in Table 5, self-efficacy ($\bar{\rho }\,$ = 0.48; H IV-1), optimism ($\bar{\rho }\,$ = 0.65; H IV-2), and conscientiousness ($\bar{\rho }\,$ = 0.40; H IV-4) are positively correlated with thriving as all 95% CIs exclude zero. However, openness ($\bar{\rho }\,$ = 0.07; H IV-3) does not correlate with thriving as the 95% CIs include zero. Therefore, with the exception of H IV1-4, H IV-1, H IV-2, and H IV-4 are supported, but H IV-3 is not supported.

### The Moderating Effect of National Culture

H V proposes that the moderating effect of national culture on the relationship between thriving and its antecedents can be found in lower-level individualistic countries and that the relationship in the countries with lower levels of individualism is stronger than in the countries with higher levels of individualism. To test this hypothesis, a random meta-regression was employed to examine all moderating effects.

As shown in Table 6, unit contextual features and individualistic culture moderate the correlations between thriving at work, autonomy (*B* = 0.006, *p* < 0.05), feedback (*B* = 0.026, *p* < 0.01), organizational justice (*B* = − 0.01, *p* < 0.01), and supportive climate (*B* = 0.006, *p* < 0.05).

**TABLE 6:** The moderating effect of national culture on the relationships between thriving at work and its antecedents.

Antecedents	Individualism		
	*k*	*B*	SE
***Unit contextual features***			
Challenge stress	6	−0.03	0.004
Hindrance stress	9	0.001	0.002
Autonomy	11	0.006*	0.003
Work control	7	−0.002	0.008
Trust	6	−0.011	0.009
Supportive climate	4	0.006*	0.003
Organizational justice	3	−0.01**	0.001
Feedback	5	0.026**	0.007
Job crafting	–	–	–
***Resources produced at work***			
Abusive supervision	–	–	–
Authentic leadership	3	0.003	0.002
Empowering Leadership	–	–	–
LMX	9	0.002	0.003
Servant leadership	3	0.011**	0.003
Transformational leadership	6	−0.09**	0.003
Positive meaning	4	0.001	0.001
Work experience	5	0.000	0.001
**Individual agentic work behaviors**			
Exploration	3	−0.01**	0.001
Task focus	6	0.003	0.003
Heedful relating	12	−0.01	0.002
**Personality traits**			
Self-efficacy	8	−0.001	0.002
Optimism	4	0.002	0.002
Openness	3	0.001	0.002
Conscientiousness	5	−0.005**	0.002

k, number of samples in the regression analysis; β, regression coefficients; *p*, the *p-*value for the coefficient. Individualism scores were coded according to the dimension data matrix on Hofstede’s website. *p < 0.05; **p < 0.01.

For leadership variables, individualistic cultures moderate the correlations between thriving and servant leadership (*B* = 0.011, *p* < 0.01), and transformational leadership (*B* = − 0.09, *p* < 0.01).

For individual agentic work behaviors, individualistic cultures moderate the correlation between thriving and exploration (*B* = − 0.01, *p* < 0.01). Finally, for personality traits, individualistic cultures moderated the correlation between thriving and conscientiousness (*B* = − 0.005, *p* < 0.01).

## Discussion

This study systematically and comprehensively meta-analyzes the relationship between antecedents and thriving at work according to the socially embedded model of thriving by Spreitzer et al. (2005) and the research of Kleine et al. (2019). It illustrates the antecedent effects of thriving, including work unit contextual features, the resources produced at work, agentic work behaviors, and personality traits. It also examines the possible influence of individualistic cultural contexts on the correlations between thriving and its antecedents.

In our meta-analysis, we found that work unit contextual features, the resources produced at work, agentic work behaviors, and personality traits have a moderate to strong effect on thriving at work.

As to the unit contextual features, most of the constructs are found to be significantly correlated with employees’ thriving at work. Among them, autonomy and work control are both positively related to the thriving of employees. Although some similarities are shared by both constructs, we believe there are still some discrepancies between these two concepts. Work control of employees refers to their control over their own work tasks and practices (Quick et al., 1990), which will benefit the working state of employees. However, autonomy focuses on more psychological experience of employees in improving their efficiency (Rhoades and Eisenberger, 2002), and will also promote employees’ thriving at work. Indeed, job crafting also reflects a certain level of autonomy and control the work of individuals. All these results further confirm the vital role of motivational factors proposed in the hygiene-motivation theory (Herzberg et al., 1959).

In terms of resources produced at work, it seems that a welcomed leader plays an important role in increasing employees’ thriving at work. According to the results, different styles of positive leadership (such as transformational leadership, authentic leadership, and empowering leadership) are believed to be positively correlated with employees’ thriving at work. The conclusions resemble the results of most studies on leadership–thriving relationships (e.g., Collins, 2014; Mortier et al., 2016; Ali et al., 2018). Although it is not our research focus in this study, the differences in these effects across different leadership styles were not confirmed. If there do exist some differences, why these distinctions occur and what the mediation effects between the leadership–thriving relationship are needed to address in the following empirical studies. We fail to predict the relationship between work experience and thriving as we expected. It occurs probably because individuals with more experience are also the aged ones, who are not easily to be thrived in their work. The limited samples in the meta-analysis were considered to be another possible reason for this issue.

Individuals who act as an agent toward their work will be more thrived at work. Consistent with the point made by Spreitzer et al. (2005), exploration, task focus, and heedful relating are reported to be positively correlated with employees’ thriving at work. Exploration indicates some exploratory behaviors (Button et al., 1996), which help individuals to be more thrived. Task focus enables individuals to be more concentrated on their tasks, and also to be more thrived (Ryan and Deci, 2000; Brown and Ryan, 2003). Meanwhile, heedful relating of individuals facilitates them to be energetic and studious ones by offering help to others and acquiring new skills (Spreitzer et al., 2005).

Personality traits (e.g., self-efficacy, optimism, and conscientiousness) are also the correlates of employees’ thriving at work. Individuals with stable positive personality traits are more likely to be thriving at work. This was consistent with the results of those studies that focused on personality-related predictors of thriving at work (Ren et al., 2015). However, contrary to our expectations, openness was not related to thriving at work. The insignificant result might be also due to a small sample in the meta-analysis.

In addition, we found that the relationships between thriving and antecedents (autonomy, feedback, supportive climate, and servant leadership), which were supposed to provide employees with support and discretion, are stronger in a higher-level individualistic country. In contrast, the relationships between employees’ thriving at work and antecedents (organizational justice and transformational leadership) that can represent the quality of the relationship between employees and colleagues, and antecedents (exploration and conscientiousness) that describe the self-characteristics of an individual are stronger in the lower-level individualistic cultures. The possible explanation might be that individuals tend to react to the information in terms of personal goals and objectives in individualistic countries, thus the enabling conditions, such as discretion and support that could help them better get job done, could more likely to encourage them to be thrived. In contrast, individuals in the collectivistic culture concerned more on the relationship with each other. Therefore, the indicators that could help them get well along with others could more likely to drive them thriving.

This study theoretically contributes to the existing literature with the following: Firstly, this research focuses on more categories of antecedents of employee thriving compared with the model of Kleine et al. (2019), which provides a comparably comprehensive review for existing empirical studies. Although the study by Kleine et al. (2019) contributed a great deal, theoretically, to recent literature, their study meta-analyzed only two categories of antecedents of employee thriving: individual characteristics and relational resources. In addition to individual characteristics, we believe the effects of unit contextual features on thriving were also important indicators for employees’ thriving at work, including those of hindrance/challenge stress, autonomy, job crafting, etc. Therefore, our study diverts the attention of following researchers from individual characteristics to a working environment when exploring the antecedents of employees’ thriving at work. We also explore the relationships between the thriving of employees and the three types of agentic work behaviors in our meta-analysis as these behaviors are regarded as an important engine of thriving in the model of Spreitzer et al. (2005).

Secondly, this research includes a greater number of indicators in each category of antecedents of employee thriving in the model described by Kleine et al. (2019) and tries its best to depict a full picture of the indicators, which may lead to employees’ thriving at work. In terms of relational resources, Kleine et al. (2019) meta-analyzed the relationships of employees’ thriving and 10 types of relational resources: heedful relating, supportive colleague behavior, workplace civility, etc. This study adds a systemic classification to these relational resources by incorporating the model of Spreitzer et al. (2005), and assigning them to leadership, positive meaning, and work experience. Such classifications ensure a better understanding for future researchers when they attempt to interpret the corresponding resources that may lead to employees’ thriving at work. Specifically, the relational resources of leadership include abusive supervision, authentic leadership, empowering leadership, LMX, servant leadership, and transformational leadership, which supplement the study of Kleine et al. (2019) with a more comprehensive analysis of the effects of various types of leadership on thriving. Additionally, a meta-analysis of the influence of positive meaning and knowledge resources (i.e., work experience) on thriving was performed, and the results demonstrate small-to-moderate positive effects.

However, with regard to personality traits, this study supplements the study of Kleine et al. (2019) of the antecedents of thriving, which included the individual characteristics of psychological capital, core self-evaluation, proactive personality, positive affectivity, negative affectivity, perceived stress, and job engagement. This study includes a meta-analysis of four types of personality traits: self-efficacy, optimism, openness, and conscientiousness. In a slight contrast to our expectations, openness does not influence thriving, whereas other types of personality traits moderately and positively influence thriving.

Thirdly, we examine the cultural differences of individualism in the relationship between antecedents and thriving at different levels of individualism. Knowledge of the influence of antecedents in different cultural contexts may assist in establishing boundary conditions for the theory of thriving. Our results are thought provoking as we find the difference of the moderating effects of individualistic culture on the relationships between employees’ thriving and variables such as autonomy, feedback, supportive climate, comparing with that between thriving and variables such as organizational justice and transformational leadership. We also encourage future research to design a very finely grained empirical study to verify the different influences of culture on the relationship between thriving at work and its different correlates.

Practically, for organization managers, this study has critical implications. Firstly, it is important that managers show a concern toward the influence of unit contextual features on employee thriving. Our results indicate that work practices and scheduled procedures, such as work autonomy, work control, job crafting, and feedback, can facilitate employee thriving by prompting managers to redesign work scopes for strengthening the employee direction, thereby increasing employee learning and vitality. Additionally, cultivating an atmosphere of respect and trust is important for thriving as our results show that trust, organizational justice, and supportive climate have significant impacts on employees. These antecedents are significant factors in cultivating a trusting and respectful climate, and can contribute to the active learning and vitality in the workplace of employees.

Secondly, another managerial implication comes from the importance of leadership. Our results show that negative leadership, such as abusive supervision, negatively influences thriving. Further, various types of positive leadership, such as authentic leadership and servant leadership, significantly and positively influence thriving. Managers should acknowledge that more employees self-actualize through the social development of modern society, and, therefore, the role of supervisors will become more quiescent by providing support and authority to employees. Autocratic leadership, including abusive leadership, is not conducive to employee growth and hinders organizational development.

Finally, although there are studies that emphasize personality traits as inherent and immutable throughout the growth of an individual, our results show that, in fact, the personality traits of self-efficacy, optimism, and conscientiousness positively influence thriving. Managers frequently use personality tests for prospective employees as positive personality traits engender thriving at work. Employees with positive personality traits actively learn and have increased vitality, leading to self-development at work.

## Limitations and Future Directions

This study has some limitations. Firstly, most of the empirical studies used in our analysis examined the antecedents of thriving at an individual level. However, fewer studies examined the high-level indicators on thriving such as group climate, inter-group communication, or intra-group communication. Furthermore, there are a limited number of studies on the influence of culture at an organizational or a national level. Therefore, we suggest that future research pays attention to the effects of high-level indicators, such as climate and culture, on employee thriving in multi-level models.

Secondly, there are a limited number of studies, which use the data from multiple sources or multiple waves. Therefore, it is not possible to analyze the moderating effects of the methodology such as the moderation of multiple raters (Zhang and Bednall, 2016). Therefore, a lot of research on thriving set one employee as a single rater. We encourage future research to employ multiple raters by using employee and colleague ratings to examine the effects of antecedents on the collective thriving of employees as individuals and relative to others. Additionally, the samples reported in most empirical studies are cross-sectional, meaning that measurement and acquiescence bias were unavoidable, thus these studies may be affected by a common method bias. Therefore, we encourage future research to collect the data from multiple waves or have used experience sampling methods to test the dynamic relationship between the different levels of antecedents and employee thriving.

Finally, in this study, existing empirical studies failed to provide a full picture of the antecedents of thriving as we could not access sufficient effect sizes to meta-analyze certain antecedent–thriving relationships. For example, we intended to meta-analyze the effects of all Big-Five personality domains on thriving, but were unable to collect sufficient effect sizes for the variables of extroversion and agreeableness. Therefore, to complete the research on the antecedents of thriving at work, we encourage future research to explore further systematic indicators of thriving.

## Conclusion

Using the socially embedded model of thriving of Spreitzer et al. (2005) and the research of Kleine et al. (2019) as starting points, we systematically and comprehensively meta-analyze the relationship between antecedents and thriving at work. Our findings suggest that there are correlations between thriving at work and its antecedents, including unit contextual features, the resources produced at work, agentic work behaviors, and personality traits. Furthermore, it is shown that cultural differences, such as individualism, play a moderate role in the influence of certain antecedents on thriving at work. This study adds substantially to our understanding of which and how antecedent variables impact thriving at work. Moreover, it makes several noteworthy contributions to the influence of individualistic culture, which acts an addition to the effects of the antecedents of thriving.

## Data Availability Statement

The raw data supporting the conclusions of this article will be made available by the authors, without undue reservation.

## Author Contributions

DL reviewed the literature, proposed the research model, and designed the study. YY conducted the literature search, proceeded with the data extraction process, and involved in the development of the manuscript. YW conducted the statistical analysis and revised the manuscript critically for important content. DL and SZ wrote the first draft of the manuscript. All authors have approved the final manuscript to be published.

## Conflict of Interest

The authors declare that the research was conducted in the absence of any commercial or financial relationships that could be construed as a potential conflict of interest.

## Publisher’s Note

All claims expressed in this article are solely those of the authors and do not necessarily represent those of their affiliated organizations, or those of the publisher, the editors and the reviewers. Any product that may be evaluated in this article, or claim that may be made by its manufacturer, is not guaranteed or endorsed by the publisher.

## Footnotes

1
https://www.hofstede-insights.com/country/china/
